# A population-based nested case control study on recurrent pneumonias in children with severe generalized cerebral palsy: ethical considerations of the design and representativeness of the study sample

**DOI:** 10.1186/1471-2431-5-25

**Published:** 2005-07-19

**Authors:** Rebekka Veugelers, Elsbeth AC Calis, Corine Penning, Arianne Verhagen, Roos Bernsen, Jan Bouquet, Marc A Benninga, Peter JFM Merkus, Hubertus GM Arets, Dick Tibboel, Heleen M Evenhuis

**Affiliations:** 1 Intellectual Disability Medicine, department of General Practice Erasmus MC, PO Box 1738, 3000 DR Rotterdam, The Netherlands; 2 Department of General Practice Erasmus MC, PO Box 1738, 3000 DR Rotterdam, The Netherlands; 3 Department of Paediatric Gastro-enterology Erasmus MC, PO Box 1738, 3000 DR Rotterdam, The Netherlands; 4 Department of Paediatric Gastro-enterology and Nutrition Academic Medical Centre / Emma's Children's Hospital, G8 217, Meibergdreef 9, 1105 AZ Amsterdam, The Netherlands; 5 Department of Paediatric Pulmonology Erasmus MC, PO Box 1738, 3000 DR Rotterdam, The Netherlands; 6 Department of Paediatric Pulmonology UMC, HP KH.01.419.0, PO Box 85590, 3508 AB Utrecht, The Netherlands; 7 Department of Paediatric Surgery Erasmus MC, Sophia Children's Hospital, PO Box 1738, 3000 DR Rotterdam, The Netherlands

## Abstract

**Background:**

In children with severe generalized cerebral palsy, pneumonias are a major health issue. Malnutrition, dysphagia, gastro-oesophageal reflux, impaired respiratory function and constipation are hypothesized risk factors. Still, no data are available on the relative contribution of these possible risk factors in the described population. This paper describes the initiation of a study in 194 children with severe generalized cerebral palsy, on the prevalence and on the impact of these hypothesized risk factors of recurrent pneumonias.

**Methods/Design:**

A nested case-control design with 18 months follow-up was chosen. Dysphagia, respiratory function and constipation will be assessed at baseline, malnutrition and gastro-oesophageal reflux at the end of the follow-up. The study population consists of a representative population sample of children with severe generalized cerebral palsy. Inclusion was done through care-centres in a predefined geographical area and not through hospitals. All measurements will be done on-site which sets high demands on all measurements. If these demands were not met in "gold standard" methods, other methods were chosen. Although the inclusion period was prolonged, the desired sample size of 300 children was not met. With a consent rate of 33%, nearly 10% of all eligible children in the Netherlands are included (n = 194). The study population is subtly different from the non-participants with regard to severity of dysphagia and prevalence rates of pneumonias and gastro-oesophageal reflux.

**Discussion:**

Ethical issues complicated the study design. Assessment of malnutrition and gastro-oesophageal reflux at baseline was considered unethical, since these conditions can be easily treated. Therefore, we postponed these diagnostics until the end of the follow-up. In order to include a representative sample, all eligible children in a predefined geographical area had to be contacted. To increase the consent rate, on-site measurements are of first choice, but timely inclusion is jeopardised. The initiation of this first study among children with severe neurological impairment led to specific, unexpected problems. Despite small differences between participants and non-participating children, our sample is as representative as can be expected from any population-based study and will provide important, new information to bring us further towards effective interventions to prevent pneumonias in this population.

## Background

Children with severe generalized cerebral palsy often have a combination of motor and intellectual disabilities. They frequently experience co-morbidity and their life expectancy is low [1-11] with respiratory disease as a main cause of death [1-3,8,10,12]. Although it is common clinical knowledge that children with neurological impairment often have respiratory problems [13-17], get hospitalised for this [18] with a major impact on their quality of life and life expectancy [14], prevalence rates have not been studied prospectively. Retrospective prevalence estimates of pneumonias range from 31% per 6 months; 38% single episodes to 19% recurrent pneumonias per year [19,20]. Although several clinical specialists presume several conditions to be risk factors for pneumonias, population-based studies on this subject are lacking. Epidemiological identification of such risk factors will bring us further towards effective interventions to prevent pneumonias.

Hypothesized risk factors of respiratory disease in children / adolescents with neurological impairment / intellectual disabilities from the literature are listed in Table 1. These factors may co-exist and interact with each other. On top of this, normal childhood factors may exist, such as asthma or passive smoking. Pneumonias can be infectious or chemical of nature. To prevent pneumonias, adequate function of the protection mechanisms of the airways is essential. But in children with severe generalized cerebral palsy this protection system is often compromised or endangered due to several conditions [14,15,20-29]

**Table 1:** Hypothesized risk factors of pulmonary disease in children with neurological impairment / intellectual disabilities

recurrent aspiration (dysphagia, gastro-oesophageal reflux) [14-16, 20, 28, 53, 54]
inefficient cough / poor cough reflex [14, 15, 28]
poor airway clearance (immobility and retained secretions) [14, 15]
respiratory muscle weakness and in-coordination [14, 15, 28]
chest wall or spinal deformities (poor pulmonary reserve) [14, 15, 28]
inadequate nutritional status (feeding problems, gastro-oesophageal reflux) [14, 15]
miscellaneous factors [2, 8, 10, 14-17]
bronchopulmonary dysplasia in preterm survivors
immune problems (Down's syndrome)
lipid aspiration in mineral oil treatment of constipation
reduced lung growth in skeletal dysplasias
normal childhood factors (e.g. asthma, passive smoking) [14, 15]
immobility [3, 10, 27, 28, 55, 56]

We hypothesize that malnutrition, dysphagia, gastro-oesophageal reflux, decreased respiratory function and constipation are the most relevant risk factors for recurrent pneumonias. Since scientific evidence for a relationship between these disorders and the occurrence of pneumonias is lacking, we aim to evaluate this in a large-scale epidemiological study. Our research questions are the following: (1) What is the prevalence of pneumonias in children with severe generalized cerebral palsy? (2) Are malnutrition, dysphagia, gastro-oesophageal reflux, decreased respiratory function and constipation risk factors for pneumonias in this group of children? The design of the study also allows us to determine the prevalence and presentation of the studied hypothesized risk factors.

This article describes the study design, diagnostic methods and the study population. Attention is paid to adaptations in the study design arising from ethical considerations as well as from the diagnostic methods required to study medical conditions in children with severe generalized cerebral palsy.

## Methods / Design

### Study design

**Figure 1:**
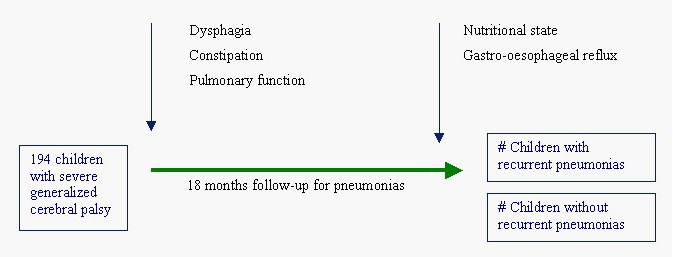
Study design. In this nested case-control study, a cohort of 194 children with severe generalized cerebral palsy is followed up for 18 months in order to record recurrent pneumonias (2 or more episodes per year). Possible risk factors are measured during the follow-up. Dysphagia, constipation and pulmonary function are diagnosed at baseline, while nutritional state and gastro-oesophageal reflux are diagnosed at the end of the study period.

This study has a nested case-control design and will be conducted in a representative group of children with severe generalized cerebral palsy, recruited through care centres (specialized day-care centres and residential facilities) and through specialized schools. In our study population, the hypothesized risk factors dysphagia, respiratory function and constipation will be assessed at baseline. However, for ethical reasons explained in the discussion paragraph, malnutrition and gastro-oesophageal reflux will be assessed at the end of the follow-up period. Cases are defined as children with recurrent pneumonias, and controls as children without pneumonias during a follow-up of 18 months. Cases and controls are matched on age, gender and GMFCS level. A duration of the follow-up period of 18 months was considered sufficient, since we defined recurrent pneumonias as 2 or more episodes within a year. The study will not interfere with common medical practice and interventions in the study population during the follow-up period. Thus, children might be diagnosed and treated by their own physicians during the course of the study. The study design is depicted in Figure 1.

### Setting

All diagnostic assessments in this study will be carried out on-site at the different care centres and specialized schools. In order to obtain a complete inclusion and therewith a representative study population, we had to keep the burden for the participants as small as possible. Hospital visits were considered an obstacle for participation. Furthermore, performing measurements in a familiar setting might improve cooperation of the children.

### Sample size

Calculating a required sample size for this study was hampered, since valid prevalence numbers of both pneumonias and most of the supposed risk factors in this population, were lacking in the literature. Prevalence numbers were estimated based on the available literature and on clinical experience. We calculated the required sample size for a univariate analysis, since the number of children required for a multivariate analysis including five separate variables will probably be quite large. In addition, we estimated that for logistical purposes a maximum number of 300 children could be included in this study. Required sample size was calculated for each possible risk factor separately, assuming a prevalence rate of recurrent pneumonias of 30% with a required power of 0.80 and an alpha of 0.05. The analysis for dysphagia, based on an estimated prevalence of dysphagia of 19% in the controls and 38% in the cases, resulted in the highest sample size (n = 260). Assuming a loss-to-follow-up rate of 13%, recruitment numbers were set to 300 participants.

### Inclusion criteria

In this study we aimed to include children (2 to 18 years), who have a combination of moderate to profound intellectual disabilities and a severe motor disability. The intellectual disability was defined as an IQ below 55 (or estimated by dividing the developmental age by the calendar age times 100). The motor disability was defined by hypertonic or hypotonic generalized cerebral palsy or a motor developmental delay to such an extent that a child can at best crawl. This corresponds to a Gross Motor Function Classification Scale (GMFCS) score IV or V [30]. These broad criteria, resulting in a heterogeneous cohort with regard to aetiology and disabilities, was chosen deliberately, because in daily practice, it is this heterogeneous group that causes a lot of concern for parents and physicians regarding the studied illnesses. Furthermore, the inclusion criteria had to be clear to non-medical personnel, to ascertain they could identify the eligible children.

### Consent procedure

We approached all children with severe generalized cerebral palsy in a certain geographical area, an important prerequisite when studying a prevalence rate, to obtain a representative sample of the total population. For pragmatic reasons, we chose an area of 50 kilometres around the cities of Rotterdam and Utrecht. We estimated that we could reach 500 children in this area. With an assumed consent rate of 0.60, this would provide the desired 300 participants. Within this area, we traced all facilities that might provide care to children and adolescents with severe generalized cerebral palsy, using the Dutch address guide for disability care. These centres were contacted and asked to participate in the study if they indeed provided care for such children. In the participating centres, parents or guardians of all children that met the inclusion criteria were informed, unless children were in a critical health status, when home situations were considered very unstable, or if parents were known to have a strong aversion to research. Information for parents was available in Dutch, English, and Turkish. For Moroccan families, a spoken introductory compact disc was available, since Berber is only a spoken language. Because gastro-oesophageal reflux can only be measured properly using an invasive method, parents had the opportunity to give consent with or without this measurement.

### Inclusion period

Of the 93 care centres and specialized schools that had been contacted, 61 provided care for one or more children with severe generalized cerebral palsy. Fifty-six of these centres agreed to participate in our study. The other centres did not cooperate due to personnel shortage and besides this, one centre also considered the burden of the study for parents, children and personnel too large.

### Participants

**Figure 2:**
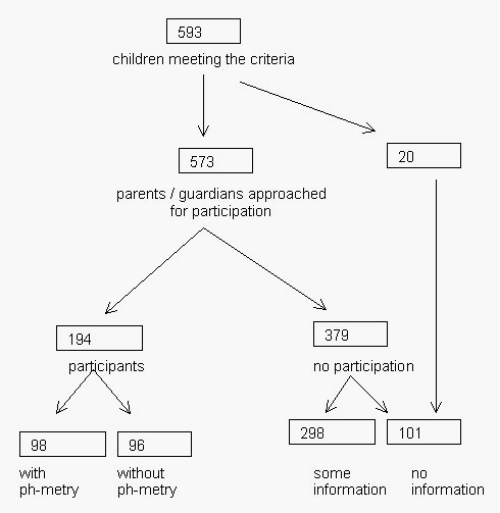
Flow chart of inclusion period. This figure depicts the inclusion of eligible children in the study from a predefined geographical area. 593 children met our inclusion criteria and parents or guardians of 573 children were informed. For several reasons, parents of 20 children were not informed. For 194 children informed consent was obtained and for 98 of those with additional consent for assessment of gastro-oesophageal reflux. For 379 children no consent was obtained. Carers of 298 of these children filled in a small questionnaire. Of 101 children no information was obtained.

Within the participating care centres and specialized schools, 593 children were eligible for participation. Parents of 573 children were informed while the parents of 9 children were not contacted based on the previously mentioned reasons and 11 were not contacted because of ineffective internal procedures of care centres. Four children, for whom consent was given, appeared not to meet our inclusion criteria at first visit and were excluded. After a prolonged inclusion period of 20 months, this resulted in the informed consent for 194 children (consent rate of 33%). Although recruitment numbers were set to 300 participants, we stopped the inclusion for practical reasons. We had included nearly 10% of the Dutch population of children with severe generalized cerebral palsy [31]. Parents of 98 children gave consent including assessment of gastro-oesophageal reflux (Figure 2). Because of the broad inclusion criteria, not all children fulfilled the strict definition of cerebral palsy [32], but all children had comparable disabilities. The different aetiologies of the disabilities of the participants are depicted in Table 2. Basic characteristics of the participants are listed in Table 3. All participating parents that gave consent preferred the questionnaires in Dutch, even when their native language was Turkish.

**Table 2:** Aetiology of disabilities

	n	%
**Congenital diseases**		
Miller Dieker Syndrome / lissencephaly	7	
corpus callosum agenesis	5	
Cornelia de Lange syndrome	2	
Walker-Warburg syndrome	2	
unspecified abnormal brain development	16	
other non progressive syndromes	6	
other chromosomal abnormalities	9	
Rett syndrome	3	
Alpers syndrome	4	
Aicardi-Goutieres syndrome	2	
other progressive syndromes	5	
other congenital diseases	4	
		
	**65**	33.5
**Pre and perinatal complications**		
perinatal asphyxia	18	
cerebral palsy e.c.i.	13	
cerebral haemorrhage	6	
intra uterine CMV infection	5	
other infections	4	
other causes	7	
		
	53	27.3
**Acquired **		
meningitis / encephalitis	5	
Trauma	3	
near drowning accident	2	
Other	2	
		
	12	6.2
**Combinations of causes**		
congenital and acquired disease	6	
congenital disease and perinatal complications	5	
perinatal and acquired	3	
perinatal and hereditary progressive	1	
		
	15	7.7
**Unknown cause**		
	25	12.8
**Missing**		
	24	12.3
		
		
	Total 194 children	

**Table 3:** Characteristics of the participants

		%	valid*
GMFCS score V		82.7	0.95
Can communicate "yes" and "no"		20.6	0.87
Can verbally communicate "yes" and "no"		3.1	0.87
Living with parents at home		81.4	1
Intentional movements	none	34.8	
	little	27.9	
	regularly	37.7	0.66
Involuntary movements	most of the day	29.6	
	regularly	35.2	
	< 2 hours a week	35.2	0.64
Seated > 3 hours / day		84.5	0.68
Standing < 30 minutes / week		38.3	0.59
Activity < 30 min / day		51.3	0.58

GMFCS = Gross Motor Function Classification Scale, *valid = fraction of the population with known information

### Representativeness

Global written information on children that did not participate was obtained from parents, care centres or specialized schools, concerning reasons for no consent, frequency of pneumonias, gastro-oesophageal reflux, body mass index and diet. To our clinical experience, parental judgement of eating skills is unreliable. Therefore we asked which food types the child received and reformulated this into a rough scale of dysphagia. Children were categorised as severe dysphagic if they received daily tube feeding, with or without additional oral food. Children with dietary restrictions (liquid, solid, ground, pureed) were categorised as having moderate dysphagia. All other children were categorised as having "no or mild" dysphagia.

Brief written information on children's characteristics was acquired for 298 of the non-participants (for 169 children from parents and for 129 children from the care centre and school personnel). Information from 101 children that were asked to participate (17%) is lacking. The main reported reasons for not participating were that parents were reluctant to any additional "hassle" with their child, mostly because of the extended medical history. Parents also considered the burden too large for themselves. Table 4 shows that the children that participate are slightly younger of age, and therewith have shorter height and lower body weight than the eligible children not participating in the study (BMI is not different between the groups). Gender is equally distributed. According to the parents' reports, the participating children have more severe dysphagia, more lower respiratory infections, and more gastro-oesophageal reflux than the non-participants.

**Table 4:** Comparison of the parent-reported characteristics between the participants and non-participants

		Non-participants		Participants	
			Valid*		valid*

Total number		379		194	
Mean Age (years)		10.6 (4.3)	0.67	8.9 (4.4)	1
Gender (% of boys)		50.2	0.7	53.1	1
Mean Height (cm)		130.3 (21.9)	0.52	124.0 (20.1)	0.91
Median Weight (kg)		28.0 [17.0]	0.59	24.7 [16.1]	0.88
Median BMI (kg/m^2^)		16.4 [4.2]	0.51	15.9 [4.0]	0.85
Dysphagia	severe (%)	27.3		37.8	
	moderate (%)	17.7		51.2	
	no / mild (%)	55.0	0.68	11.0	0.65
Lower respiratory tract infections (%)		16.9	0.68	27.3	0.45
recurrent** (%)		12.5	0.67	18.2	0.45
Reported gastro-oesophageal reflux (%)		25.1		44.3	0.72

Standard deviations are between brackets, inter quartile range is between square brackets, *valid = fraction of the population with known information, ** recurrent = two or more episodes per year, BMI = body mass index.

### Diagnostic methods

Diagnostic methods had to be chosen with great care. Because all assessments are performed on-site, diagnostic methods should be ambulatory available. Moreover, standard methods are often not feasible, due to the severity of the handicaps of these children, and the required level of cooperation. The Dutch ethics committee also demanded methods to be non invasive, if possible.

### Pneumonia

In clinical practice, pneumonia is diagnosed based on a chest X-ray together with symptoms and signs. In the present study however, we needed to use a definition that could be used without requiring extra diagnostic procedures. A previous study showed that retrospective examination of medical files was not accurate for detection of pneumonias [33]. Therefore, the research team agreed upon the following definition for an episode of pneumonia: fever (> 38.5°C, or 1,5°C above basal temperature) during more than 24 hours, likely due to a pneumonia, characterized by: (increase of) dyspnoea (tachypnoea, use of assistant respiratory muscles, wheezing) during the last 6 hours, and/or (increase of) hyper secretion of mucus, and/or, tachypnoea and regular coughing. In addition, no other explanation for fever (such as middle ear infection or a urinary tract infection) should be present. Because this is a population-based study, participating children all have their own treating physicians. To limit the number of people that are involved in gathering data on pneumonias, parents were asked to complete a questionnaire whenever their child has a fever and airway symptoms. If a physician is contacted, parents ask him or her to fill in a questionnaire for physicians. Every 4 months, parents will be reminded to complete the questionnaires if their child was ill.

### Respiratory function

The gold standard technique, spirometry, is not feasible for this population due to the low developmental age and motor disabilities [34]. We will measure respiratory function using the interruption technique. A reversibility test will be done using Salbutamol. This is a well-studied technique that is commonly used in infants. Reliability is high and the ambulatory equipment is commercially available. [35-40] In addition, reference values are available for children. [34,41-44]

### Dysphagia

In a hospital setting, aspiration can be assessed with videofluoroscopy. Since this technique is not ambulatory available, we will assess severity of dysphagia instead of aspiration. For this epidemiological study we have chosen a standardized observation method: the Dysphagia Disorder Survey (DDS) / Dysphagia Management Staging Scale (DMSS). This method has been developed especially for people with developmental disabilities [45]. We will combine this method with cervical auscultation and measurements of oxygen saturation, to increase accurateness of the observation.

### Constipation

To assess constipation, we will use structured parental interviews, a two-week defecation diary and a one-week diary on food intake. This will be combined with a physical examination of the abdomen and the anal area [46]. In clinical practice, the physical examination also includes a digital rectal palpation to assess faecal impaction. However, this was considered too invasive by the ethics committee.

### Nutritional state

To assess nutritional state, we will use classical anthropometry in accordance with Gerver & de Bruin [47] and single frequency Bioelectric Impedance Assessment (BIA) [48].

### Gastro-oesophageal reflux

Gastro-oesophageal reflux will be assessed using the gold standard method, 24-hour pH-metry [49]. However, to make this test feasible for on-site measurements, catheter placement will not verified by X-ray, but the step-up method will be used [50,51].

### Analysis and statistics

Incidence of pneumonia will be studied prospectively and the prevalence of the hypothesized risk factors will be studied cross-sectionally. The association between the hypothesized risk factors and recurrent pneumonias will be assessed using logistic regression. A Poisson regression will be used to analyse their influence on pneumonia incidence. In these analyses, only the cases and their controls will be used. The required number of controls will depend on the number of cases. P-values less than 0.05 will be considered significant.

### Ethical approval

Ethical approval was obtained (P02.0188C) from the national ethics committee (The Central Committee on Research Involving Human Subjects). Care centres and specialized schools formally consented to participate. Parents or legal guardians gave informed consent, with or without consent for gastro-oesophageal reflux. Because gastro-oesophageal reflux can only be measured properly using an invasive method, parents had the opportunity to give consent with or without this measurement.

## Discussion

Designing and conducting an epidemiological study in children with severe generalized cerebral palsy is associated with characteristic difficulties. Even though we have considerable experience with research through care organisations [52], the initiation of this first study in children lead to specific, not always anticipated, problems, which caused a substantial delay. In the present study several obstacles needed to be overcome, which will most likely be encountered in future studies as well. This started with the design of a realistic, ethically acceptable study, including the choice of feasible diagnostic assessment methods and was followed by the recruitment of a representative cohort. In addition, one should bear in mind that on-site measurements and therewith inclusion through care centres (specialized day-care centres and residential facilities) and specialized schools can jeopardise timely inclusion due to potential lengthy procedures.

### Dealing with encountered obstacles

Designing the study was complicated by ethical issues, which were resolved by a limited concession in the study design. In standard (nested) case-control studies, hypothesized risk factors are determined at baseline. In the present study, indeed, we will determine respiratory function, constipation and dysphagia at the start of the study, as risk factors. However, gastro-oesophageal reflux and malnutrition are disorders that are likely to cause a considerable loss of quality of life, apart from their possible effects on pneumonias, and both can easily be treated. Therefore, it was considered ethically unacceptable to determine the presence of these conditions at the start of the follow-up and then postponing treatment until the study would be finished. For that reason, we decided to perform the diagnostic tests for these conditions at the end of the follow-up period. This theoretically reduces the power of the analysis, but this reduction is relative since both conditions have a chronic character. We consider this design ethically acceptable, even though we purposely will not assess gastro-oesophageal reflux and nutritional state at baseline, because we will not interfere with common medical practice. Therefore, medical diagnosing and treatment of these disorders will not be hampered.

To conduct this study, a group of children with recurrent pneumonias needed to be identified prospectively. It would make sense to do this retrospectively. However, a previously conducted pilot study indicated that medical records, even when combined with interviews of paediatricians and intellectual disability physicians, provided incomplete and therefore unreliable information on pneumonias in these children [33].

Getting informed consent of the carers of all eligible children in a geographical area within a reasonable time span was difficult. Firstly, there was no clear registration of the centres that provide care for this specific population in the Netherlands, which resulted in a search amongst a range of organisations. Secondly, centres all had their own procedure to decide on cooperation with a study, often including management, medical staff, other personnel, parent boards and ethics committees. In some centres no standard procedure existed, since they had never been asked to participate in a study before. Thirdly, the national ethics committee considered this study as a multi-centre study and required a consent-form from each centre in advance of their final approval. Although this procedure works well in studies with 2 or 3 participating hospitals, for the present study it meant that 56 centres needed to decide on participation in advance. The resulting delay was a new and unsatisfying experience for the national ethics committee as well. Fourthly, privacy regulations lead to great dependence on willingness and organizational skills of the participating centres. The selection of eligible children had to be done by care centre personnel, and information brochures were sent while researchers were blinded for names and addresses. Despite these encountered difficulties, we have approached a representative sample of children with severe generalized cerebral palsy.

All diagnostic measurements should be ambulatory available and require no active cooperation. Therefore, not all diagnostic methods in this study are "gold-standard" methods. To date, only few diagnostic tests are available, validated for this specific population. Some diagnostic tests used in the present study are applied for the first time in this population, resulting in valuable feasibility data for future validation studies. Since ethical regulations also required methods to be non-invasive when possible, assessment of constipation need to be done without the rectal digital examination, which will therefore provide less information in comparison to the normal diagnostic procedure.

To ensure that people of different nationalities participate in a prevalence study, information needs to be provided in several languages. However, our experience is that there is no need for translated written information brochures and questionnaires. A spoken introduction on compact disc can provide an introduction and interested parents will ask a family member for translation of the brochure and questionnaires.

Finally, the inclusion period was stopped before target sample size was reached, due to delay because of practical reasons discussed above. By the end of our inclusion period, almost a quarter of the children with severe generalized cerebral palsy in the Netherlands had been approached and nearly 10% of the Dutch population of these children participates. Even with less power than desired, this study will be able to put a subject on the map that got little attention up to now.

### Representativeness

To stay close to clinical practice, we used inclusion criteria based on disabilities rather than on aetiology, resulting in a heterogeneous group of children. Obviously, this might also cause more heterogeneity of the results.

The participating children are slightly younger of age than the eligible children that did not participate. However, we do not regard an age difference of less then 2 years with a standard deviation of over 4 years, as a clinical relevant discrepancy. Height and weight differences can be explained by age, since BMI is not different between both groups. A relevant discrepancy does seem to be present between the groups with regard to the reported severity of dysphagia, the frequency of lower respiratory tract infections and the presence of gastro-oesophageal reflux. We assume that the parents of the children with more severe health problems were more likely to recognize the health issues of their child in the information brochure and therefore decided to participate more often. Since swallowing strongly depends on motor skills, it seems likely that participants have poorer motor skills in general then the non-participants. Another part of the discrepancy might be explained by the selection of non-eligible children by staff of the centres. On first visit, we had to exclude four children whose motor or intellectual skills were of a higher level than those defined by our inclusion criteria. This might also have been the case in the group that did not consent to participate. Because of the slight discrepancies in characteristics, the final results, especially prevalence rates, have to be interpreted with caution. Despite the discrepancies, our sample is as representative as can be expected in population-based research.

### Implication for future studies

Preventive medicine needs to play a major role in the healthcare for children with severe neurological impairment. Consequently, intervention studies are needed in which effects can be measured in a valid and reproducible way, and reference values need to be established. As in any discipline, intervention studies should be based on epidemiological data. To avoid complex epidemiological studies, a health register seems to be a requisite. In such a registry, data on health status, diagnostic assessments and applied medical treatments of children with severe neurological impairment should be recorded. This would also enable specialists to combine knowledge and to monitor trends.

For every study question, one should contemplate on the choice between diagnostic assessments in hospital or on-site. When a representative cohort of children with severe generalized cerebral palsy is required, one should perform a community-based study to keep the burden low and therewith the consent rate as high as possible, but one can expect to encounter the discussed obstacles. The main disadvantage of a hospital-based study is that a selective population will be recruited, even when performed through an outpatient clinic. Furthermore, one should consider that feasibility of diagnostic assessments might be better on-site, due to the fact that the setting is familiar to the child. On the other hand, in hospital-based studies, logistics are less complicated and hospital assessments, such as X-rays, are easily applied.

In conclusion, this study will fill in some of the lacunas in the knowledge of the health status of these children such as prevalence numbers of several health conditions, associations with recurrent pneumonias. It will also provide new information on the diagnostic tools available for these children, and provide experience in performing scientific studies in this specific field.

## List of abbreviations

BIA Bioelectric Impedance Assessment

BMI Body Mass Index

IQ Intelligence Quotient

## Competing interests

The author(s) declare that they have no competing interests.

## Authors' contributions

RV contributed to the design of the study, coordinated the inclusion and data acquisition, acquired and analysed data and wrote the article. EACC contributed to the acquisition of data and has been involved in revising the article critically for important intellectual content. CP was responsible for the conduct of the study and helped to draft the manuscript. HGMA has contributed to the design and had been involved in revising the article critically for important intellectual content. AV, RB, JB, MAB, PJFMM contributed to the conception of the study and design and have been involved in revising the article critically for important intellectual content. DT participated in the design and conception of the study and helped to draft the manuscript. HME was the initiator of the study, participated in its design and helped to draft the manuscript. All authors read and approved the final version of the manuscript.

## Pre-publication history

The pre-publication history for this paper can be accessed here:


